# In Vivo Tau and Neurodegeneration Imaging in a Family With the Presenilin 1 Met146Leu Pathogenic Variant

**DOI:** 10.1212/WNL.0000000000210103

**Published:** 2024-11-25

**Authors:** Cecilia Boccalini, Alessandra Dodich, Max Scheffler, Valentina Laganà, Enrico Fratto, Giovanni B. Frisoni, Amalia Cecilia Bruni, Rosanna Colao, Daniela Perani, Valentina Garibotto

**Affiliations:** From the Laboratory of Neuroimaging and Innovative Molecular Tracers (NIMTlab) (C.B.), Geneva University Neurocenter and Faculty of Medicine, University of Geneva, Switzerland; Center for Mind/Brain Sciences (A.D.), CIMeC, University of Trento, Italy; Division of Radiology (M.S.), Geneva University Hospitals, Switzerland; Department of Primary Care (V.L., A.C.B., R.C.), Regional Neurogenetic Centre, ASP Catanzaro, Lamezia Terme; Institute of Neurology (E.F.), Department of Medical and Surgical Sciences, Magna Graecia University, Catanzaro, Italy; Geneva Memory Center (G.B.F.), Geneva University Hospitals, Switzerland; Nuclear Medicine Unit (D.P.), San Raffaele Hospital, Milan; Vita-Salute San Raffaele University (D.P.), Milan, Italy; and Division of Nuclear Medicine and Molecular Imaging (V.G.), Geneva University Hospitals, Switzerland.

## Abstract

**Objectives:**

We investigated tau and neurodegeneration patterns and clinical phenotypes in carriers of a specific pathogenic variant in the PSEN1 gene and 1 nonaffected relative.

**Methods:**

We included 3 symptomatic carriers of the c.436 A>C, p.Met146Leu, NM_000021.4, rs63750306 variant in the PSEN1 gene, pathogenic for autosomal dominant Alzheimer disease (AD), 1 asymptomatic carrier of the same variant, and 1 noncarrier, all belonging to the same “N” family. All subjects underwent clinical evaluations, ^18^F-flortaucipir-PET, and MRI. ^18^F-fludeoxyglucose-PET was available for 3 cases.

**Results:**

All symptomatic carriers showed advanced AD tau patterns. Symptomatic female carriers presented an earlier age at onset and more pronounced tau pathology in temporoparietal and frontal regions than male carriers, at comparable disease severity and duration. The presymptomatic male carrier showed a negative tau scan 4 years before symptom onset. MRI showed no severe cortical and hippocampal atrophy in all individuals. Brain metabolism showed neurodegeneration patterns typical of AD in symptomatic carriers.

**Discussion:**

In PSEN1 Met146Leu variant carriers, high cortical tau load, without significant atrophy, was present during early memory deficits. In the asymptomatic phase, all biomarkers were negative. More pronounced tau pathology in female than male individuals highlights the need to investigate sex differences in autosomal dominant AD.

## Introduction

Alzheimer disease (AD) is characterized by extracellular β-amyloid (Aβ) deposits and intraneuronal neurofibrillary tau tangles.^1^ Autosomal dominant AD (ADAD), caused by variants in the presenilin (PSEN1, PSEN2) and Aβ precursor protein (APP) genes, provides a model for the chronologic study of pathophysiologic changes in AD, because of its almost complete penetrance. The study of individuals carrying variants in these genes at different ages and over time allows to study the temporal evolution of the pathologic processes of AD.^2^ Studies performed on populations of ADAD variant carriers demonstrated abnormal Aβ deposits, more than 10 years before the onset of symptoms.^3-6^ Previous studies showed no significant tau deposition in 20 asymptomatic carriers with different pathogenic variants in PSEN1, PSEN2, and APP,^4^ although increases may be observed in specific regions (such as the precuneus) in carriers near symptom onset^6,7^ or in the medial temporal lobe 6 years before disease onset.^3^

In this study, we investigated in vivo the presence and distribution of tau pathology and neurodegeneration using PET and MRI in individuals belonging to the “N” family.^8^ This is a large pedigree of Calabrian origin harboring the PSEN1 c.436 A>C, p.Met146Leu, NM_000021.4, rs63750306 variant, classified as pathogenic for ADAD according to the American College of Medical Genetics and Genomics guidelines.^9^ The “N” family is named after “Nicastro,” the Calabrian town giving the origins to the first family proband.^8,10^

## Methods

### Subjects

The participants were recruited from the Regional Centre for Neurogenetics in Lamezia Terme (Italy). They all belong to the previously described “N” family. We included 5 subjects who underwent imaging examinations, genetic testing, and full clinical assessment. All patients were informed about their genetic status after an informed consent was signed by them or their next of kin.

Studies were conducted following the principles of the Declaration of Helsinki and the International Conference on Harmonization Good Clinical Practice, and each subject provided voluntary written informed consent.

### Clinical Assessment

Cognitive status was assessed through the Mini-Mental State Examination at different time points (at the onset of first detectable subjective symptoms and/or clinical cognitive signs, imaging time point, and last follow-up). An extensive neuropsychological assessment was performed at imaging time, encompassing evaluation of memory, language, executive function, and visuospatial skills. The clinical syndromes of mild cognitive impairment (MCI) and dementia were defined in compliance with the *Diagnostic and Statistical Manual of Mental Disorders, 5th Edition* criteria for mild and major neurocognitive disorder.^11^

### Imaging Assessment

Different imaging investigations were performed at different time points for the 5 participants and are described in eMethods and in the Table. We used visual and semiquantitative procedures to define subjects as positive or negative for each imaging biomarker, as described in eMethods.

**Table:** Clinical Characteristics of Study Participants

Case	PSEN1 variant^a^	Sex	Education	Age at clinical onset	Presenting symptoms	MMSE at onset	Age at imaging time	Clinical symptoms at imaging time	Clinical stage at imaging time	MMSE at imaging time	Tau-PET pattern (SUVR cut-off = 1.24)	MRI AD cortical thickness (cutoff = 2.57) and HPV (cut-off = 0.0021)	FDG-PET pattern	Age at last follow-up	MMSE at last follow-up
Case 1	Yes (M146L)	Female	8	38	Memory deficits	27/30	47	Amnestic deficits in learning and storage, visuoconstructive abilities and verbal fluency. behavioral alterations (apathy, irritability)	Dementia	22/30	Positive: Braak 5 SUVR = 2.09	Cortical thickness = 2.76 HPV = 0.0015	Temporoparietal and frontal hypometabolism	52	9/30
Case 2	Yes (M146L)	Female	18	34	Memory deficits	30/30	37	Isolated delayed recall verbal memory dysfunctions and no facilitation of semantic cue	MCI	30/30	Positive: Braak 5 SUVR = 1.79	Cortical thickness = 2.95; HPV = 0.0028	NA	42	23/30
Case 3	Yes (M146L)	Male	8	45	Temporal disorientation, calculation deficit	20/30	48	Memory deficits, mild behavioral alterations	MCI	25/30	Positive: Braak 5 (left lateralized) SUVR = 1.28	Cortical thickness = 3.00; HPV = 0.0028	Temporoparietal and frontal hypometabolism (left lateralized) (4 y later than tau-PET and concomitant to the last clinical FU)	52	7/30
Case 4	Yes (M146L)	Male	10	43	Memory and verbal fluency deficits	26/30	39	Asymptomatic	CU	30/30	Negative SUVR = 1.09	Cortical thickness = 2.88; HPV = 0.0031	Hypometabolism in the precuneus, posterior and anterior cingulate (4 y later than tau-PET and concomitant to the last clinical FU)	44	26/30
Case 5	No	Female	16	NA	Asymptomatic	NA	34	Asymptomatic	CU	30/30	Negative SUVR = 1.13	Cortical thickness = 3.01; HPV = 0.0029	NA	39	Asymptomatic

Abbreviations: AD = Alzheimer disease; CDR = Clinical Dementia Rating; CU = cognitively unimpaired; FDG = fludeoxyglucose; FU = follow-up; HPV = hippocampal volume; MCI = mild cognitive impairment; MMSE = Mini-Mental State Examination; NA = not applicable; SUVR = standardized uptake value ratio.

a PSEN1 c.436 A>C, p.Met146Leu, NM_000021.4, rs63750306.

## Results

The individual clinical and biomarker characteristics of the 3 symptomatic carriers of the sPSEN1 gene variant (c.436 A>C, p.Met146Leu; cases 1, 2, 3), 1 asymptomatic carrier of the same variant (case 4), and 1 noncarrier (case 5) are detailed in the Table. None carried the APOE ε4 allele, and Aβ status was available and positive for symptomatic cases 1 and 2. Two of the 3 symptomatic carriers (cases 1 and 2) were characterized by amnesic MCI at onset and the other (case 3) by multidomain MCI with deficits in episodic memory and executive and visuoconstructive functions. Despite the family history, case 5 is a stable asymptomatic noncarrier with all imaging biomarkers resulting negative. All symptomatic carriers (cases 1, 2, 3) showed advanced tau patterns (Braak 5), involving lateral temporal, parietal, and frontal regions (Figure). The medial temporal lobe was involved only in case 2. Semiquantitative standardized uptake value ratio (SUVR) analysis showed significant uptake in the 2 female symptomatic carriers (cases 1 and 2), whereas the male symptomatic carrier (case 3) presented higher SUVR values in the left hemisphere, slightly above the threshold (Figure). The typical AD temporoparietal hypometabolic pattern, also involving the frontal cortex, was less extended in symptomatic case 1 (MCI) than in symptomatic case 3 showing a more diffuse pattern after 4 years of follow-up, when he had a dementia diagnosis. MRI at the time of tau-PET showed no presence of significant AD neurodegeneration, except for symptomatic case 1 who showed hippocampal atrophy slightly below the threshold of normality. The asymptomatic male carrier (case 4) had negative tau and MRI imaging at inclusion. After 4 years, he converted into multidomain MCI with the ^18^F-fludeoxyglucose (FDG)-PET showing bilateral hypometabolism in the precuneus, posterior and anterior cingulate gyri, and posterior parietal cortex.

**Figure:**
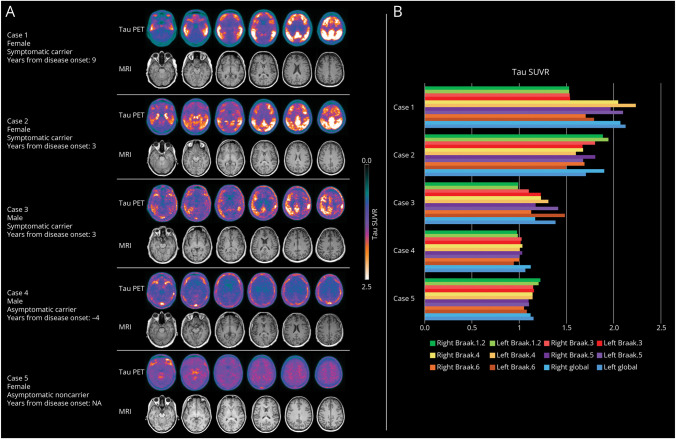
Tau and Neurodegeneration Patterns In Panel A, axial images of ^18^F-flortaucipir and MRI T1 images are shown for each study participant. ^18^F-flortaucipir PET and MRI T1 images were acquired the same day or within 2 days. The color bar shows SUVR ranges. In Panel B, the histogram shows the regional SUVRs for each participant. SUVR = standardized uptake value ratio.

## Discussion

This study describes tau accumulation, hypometabolic, and atrophy patterns in the brains of PSEN1 c.436 A>C, p.Met146Leu pathogenic variant carriers. There are limited tau-PET studies in PSEN1 gene variants^3,4,6,7,12^ and none concerning the specific Met146Leu variant. The latter is known to lead to young onset with presenting symptoms spanning from the classic AD cognitive profile (memory loss, time or spatial disorientation), as shown in case 3, to frontal behavioral profiles including apathy and irritability, as reported here in case 1.^8^ All symptomatic carriers of our sample clinically worsened and converted to dementia within approximately 8 years from onset with individual variability (Table). In a cohort of PSEN1 E280A carriers, the median time of progression from asymptomatic to pre-MCI was 4 years (95% CI 2–8), from pre-MCI to MCI was 6 years (4–7), from MCI to dementia was 5 years (4–6), and from dementia to death was 10 years (9–12).^13^ Although the mean age at onset in individuals with ADAD with PSEN1 c.436 A>C, p.Met146Leu is around 40 years, and carriers are destined to develop the disease by the end of the fifth decade,^8^ our cohort includes a 39-year-old asymptomatic male carrier (case 4) who showed a completely negative tau-PET scan also confirmed by semiquantification analysis. This result seems in line with no significant tau deposition found in presymptomatic ADAD carriers from the DIAN studies.^4,7^ Instead, all symptomatic carriers showed high uptake of ^18^F-flortaucipir in AD-related regions (Figure). According to the temporal sequence of biomarker progression,^1^ all cases did not show relevant neurodegeneration at the time of tau-PET. The global tau SUVR was particularly high in cases 1 and 2, the 2 female carriers, whereas the tau load was low and left lateralized in case 3, the male symptomatic carrier. Sex differences investigated in sporadic AD reported female individuals to have more pronounced neurodegeneration and tau pathology, as measured by CSF,^14^ PET imaging,^15^ and postmortem neuropathology. However, limited evidence exists on the role of sex in ADAD,^16-20^ and even less including tau-PET. A recent study did not find differences in plasma tau phosphorylated at threonine 217 among PSEN1 E280A variant carriers in preclinical and clinical stages; however, female carriers had a greater rate of neurodegeneration than male carriers as the disease progressed.^16^ The results in our small case series suggest a greater susceptibility to AD pathology in female Met146Leu carriers in terms of higher tau load intensity and younger age at onset than the male carrier.

Among the study limitations, CSF AD biomarkers were not measured, and amyloid status and ^18^F-FDG-PET were available in a limited number of cases and at varying time points.

In summary, ADAD due to c.436 A>C, p.Met146Leu seems to be characterized by pronounced tau pathology and reduced metabolism in temporoparietal and frontal regions, in the early prodromal symptomatic phase, and not yet in the presymptomatic stage. Tau pathology seems more pronounced in female than male individuals, even if further investigation of sex-specific differences in large ADAD cohorts is needed to elucidate the mechanisms.

## Appendix Authors

**Table:**

Name	Location	Contribution
Cecilia Boccalini, PhD	Laboratory of Neuroimaging and Innovative Molecular Tracers (NIMTlab), Geneva University Neurocenter and Faculty of Medicine, University of Geneva, Switzerland	Drafting/revision of the manuscript for content, including medical writing for content; study concept or design; analysis or interpretation of data
Alessandra Dodich, PhD	Center for Mind/Brain Sciences, CIMeC, University of Trento, Italy	Drafting/revision of the manuscript for content, including medical writing for content; major role in the acquisition of data
Max Scheffler, MD	Division of Radiology, Geneva University Hospitals, Switzerland	Drafting/revision of the manuscript for content, including medical writing for content; major role in the acquisition of data
Valentina Laganà, PsyD	Department of Primary Care, Regional Neurogenetic Centre, ASP Catanzaro, Lamezia Terme, Italy	Drafting/revision of the manuscript for content, including medical writing for content; major role in the acquisition of data
Enrico Fratto, MD	Institute of Neurology, Department of Medical and Surgical Sciences, Magna Graecia University, Catanzaro, Italy	Drafting/revision of the manuscript for content, including medical writing for content; major role in the acquisition of data
Giovanni B. Frisoni, MD	Geneva Memory Center, Geneva University Hospitals, Switzerland	Drafting/revision of the manuscript for content, including medical writing for content; major role in the acquisition of data
Amalia Cecilia Bruni, MD	Department of Primary Care, Regional Neurogenetic Centre, ASP Catanzaro, Lamezia Terme, Italy	Drafting/revision of the manuscript for content, including medical writing for content; major role in the acquisition of data
Rosanna Colao, MD	Department of Primary Care, Regional Neurogenetic Centre, ASP Catanzaro, Lamezia Terme, Italy	Drafting/revision of the manuscript for content, including medical writing for content; major role in the acquisition of data
Daniela Perani, MD	Nuclear Medicine Unit, San Raffaele Hospital, Milan; Vita-Salute San Raffaele University, Milan, Italy	Drafting/revision of the manuscript for content, including medical writing for content; study concept or design; analysis or interpretation of data
Valentina Garibotto, MD	Division of Nuclear Medicine and Molecular Imaging, Geneva University Hospitals, Switzerland	Drafting/revision of the manuscript for content, including medical writing for content; major role in the acquisition of data; study concept or design; analysis or interpretation of data
